# Anti-Inflammatory Activity of Essential Oil from *Zingiber ottensii* Valeton in Animal Models

**DOI:** 10.3390/molecules27134260

**Published:** 2022-07-01

**Authors:** Wisit Thitinarongwate, Wutigri Nimlamool, Parirat Khonsung, Raktham Mektrirat, Puongtip Kunanusorn

**Affiliations:** 1 Department of Pharmacology, Faculty of Medicine, Chiang Mai University, Chiang Mai 50200, Thailand; wisit.vetcmu@gmail.com (W.T.); wutigri.nimlamool@cmu.ac.th (W.N.); wparirat@yahoo.com (P.K.); 2 Graduate School, Chiang Mai University, Chiang Mai 50200, Thailand; 3 Department of Veterinary Biosciences and Public Health, Faculty of Veterinary Medicine, Chiang Mai University, Chiang Mai 50100, Thailand; raktham.m@cmu.ac.th

**Keywords:** *Zingiber ottensii* Valeton, essential oil, anti-inflammation, histological examination, COX-2, PGE_2_, TNF-α, rats

## Abstract

*Zingiber ottensii* (ZO) Valeton, a local plant in Northern Thailand, has been widely used in traditional medicine. Many studies using in vitro models reveal its pharmacological activities, including the anti-inflammatory activity of ZO essential oil, extracted from ZO rhizomes. However, the scientific report to confirm its anti-inflammatory activity using animal models is still lacking. The present study aimed to evaluate the anti-inflammatory activity and explore the possible mechanisms of action of ZO essential oil in rats. The results revealed that ZO essential oil significantly reduced the ear edema formation induced by ethyl phenylpropiolate. Pre-treatment with ZO essential oil significantly reduced the carrageenan-induced hind paw edema and the severity of inflammation in paw tissue. In addition, pre-treatment with ZO essential oil exhibited decreased COX-2 and pro-inflammatory cytokine TNF-α expression in paw tissue, as well as PGE_2_ levels in serum. On this basis, our study suggests that ZO essential oil possesses anti-inflammatory activity in animal models. Its possible mechanisms of action may involve the inhibition of TNF-α expression as well as the inhibition of COX-2 and PGE_2_ production. These findings provide more crucial data of ZO essential oil that may lead to new natural anti-inflammatory product development in the future.

## 1. Introduction

Inflammation is an immunological response of the body against mechanical injury, burns, allergens, and noxious stimuli [1]. This defense mechanism involves the migration of leukocytes, such as neutrophil, monocyte, and macrophage; production of inflammatory mediators, such as serotonin, bradykinin, histamine, prostaglandin E_2_ (PGE_2_), and nitric oxide; and production of pro-inflammatory mediators, such as tumor necrotic factor-alpha (TNF-α) and interleukin-1,6 (IL-1,6), to eliminate and limit any further injuries to prevent host tissue damage and initiate tissue repair [2]. Persistent unresolved inflammation can lead to various inflammatory disorders, such as atherosclerosis, diabetes, rheumatoid arthritis, inflammatory bowel disease, cancer, Alzheimer’s disease, and obesity [3,4].

Non-steroidal anti-inflammatory drugs (NSAIDs) such as ibuprofen, naproxen, and indomethacin are commonly prescribed medications for treating inflammation, fever, and pain by inhibition of the cyclooxygenase (COX) enzyme that affects the conversion of arachidonic acid to prostaglandins (PGs) [5]. However, NSAIDs have many side effects with long-term medication, such as gastrointestinal lesions, bleeding, and peptic ulcers [6,7,8]. The findings of new and effective natural products exhibiting anti-inflammation with safety and fewer adverse effects will be the alternative way for patients.

Plants in the Zingiberaceae family are widely distributed in the tropical and subtropical areas in Southeast Asia. Their rhizomes have been extensively used as food, spices, and in traditional medicine [9,10,11]. Although studies about the anti-inflammatory activity of the rhizomes of these plants have been reported [12,13], the confirmation of this activity in some plants in this family is still lacking.

*Zingiber ottensii* (ZO) Valeton or “Phlai dum” (in Thai), a local plant in Northern Thailand, is a medicinal plant that belongs to the Zingiberaceae family. Leaves and rhizomes of this plant have many traditional uses in Southeast Asia, including Thailand, Indonesia, and Malaysia, for curing peptic ulcers, stomachache, constipation, lumbago, myalgia, wounds, sprain, and bruising [14,15]. The major phytochemical compounds of ZO essential oil, extracted from ZO rhizomes, are a group of terpenes, with zerumbone as a major compound [15,16]. Many studies using the in vitro models reveal the anticancer, antidiabetic, antimicrobial, and anti-inflammatory activities of ZO essential oil [17,18,19,20]. However, the scientific reporting to confirm its anti-inflammatory activity using animal models is still lacking. Thus, the present study aimed to evaluate the anti-inflammatory activity using the well-known animal models and explore the possible mechanisms involved in the anti-inflammatory activity of ZO essential oil from rhizomes of ZO grown in Thailand. The results of this study provide additional necessary information of ZO essential oil that strengthens the possibility of ZO essential oil being developed into new herbal health products/medicines as alternative ways for anyone who denies using modern medicines in the future.

## 2. Results

### 2.1. Effect of Zingiber ottensii (ZO) Essential Oil on Ethyl Phenylpropiolate (EPP)-Induced Ear Edema in Rats

**Figure 1:**
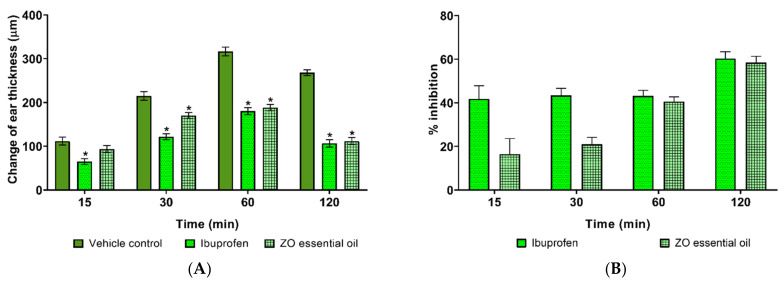
Effects of ZO essential oil and ibuprofen on EPP-induced ear edema in rats. (**A**) Mean changes in ear thickness (µm) and (**B**) the percentages of ear edema inhibition after pretreating with ZO essential oil (3 mg/ear) or ibuprofen (1 mg/ear) at different time points. Data represent the mean ± SEM (*n* = 6). * Denotes significant differences (*p* < 0.05) compared to the control group.

The effects of ZO essential oil on inflammation in the ear edema induced by EPP of rats were investigated. The mean change of ear edema thickness is presented in Figure 1A. ZO essential oil at the dose of 3 mg/ear significantly reduced ear edema formation at 30, 60, and 120 min post-induction by 45.00, 128.40, and 156.60 µm, respectively, while ibuprofen can significantly reduce ear edema formation at 15, 30, 60, and 120 post-induction by 46.70, 93.30, 136.70, and 161.60 µm, respectively, (*p* < 0.05). The percentages of inhibition at 15, 30, 60, and 120 min post-induction of ZO essential oil (16.42, 20.93, 40.53, and 58.39) and ibuprofen (41.79, 43.41, 43.16, and 60.25) groups are shown in Figure 1B.

### 2.2. Effect of ZO Essential Oil on Carrageenan-Induced Hind Paw Edema in Rats

**Figure 2:**
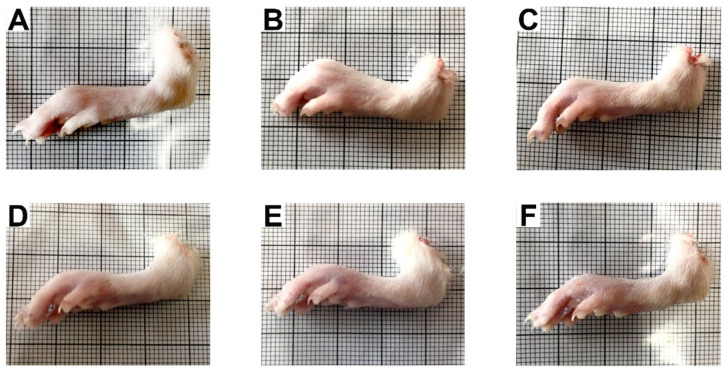
Gross pathological changes of hind paws of rats pretreated with vehicle (0.9% saline), ibuprofen (100 mg/kg), or ZO essential oil at different doses at 6 h after carrageenan injection. (**A**) Normal control group (no carrageenan injection); (**B**) control group; (**C**) ibuprofen group; (**D**–**F**) 200, 400, and 800 mg/kg of ZO essential oil groups.

**Figure 3:**
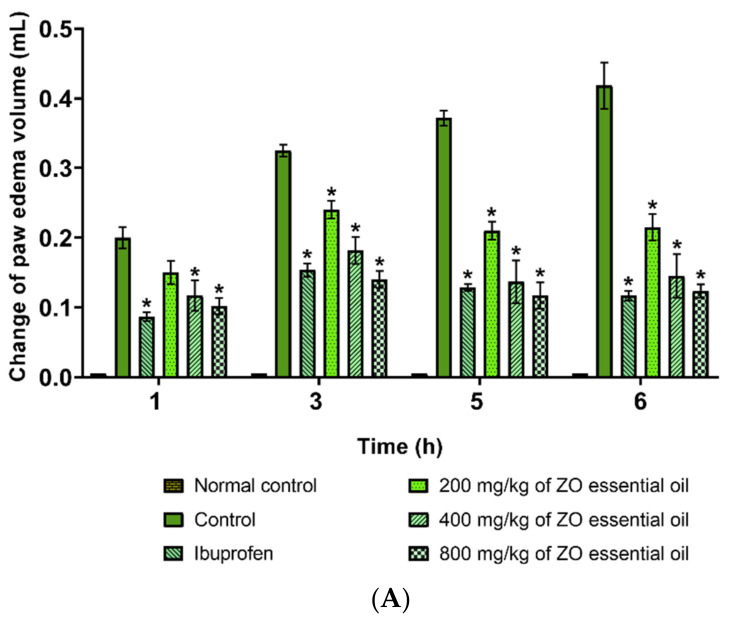

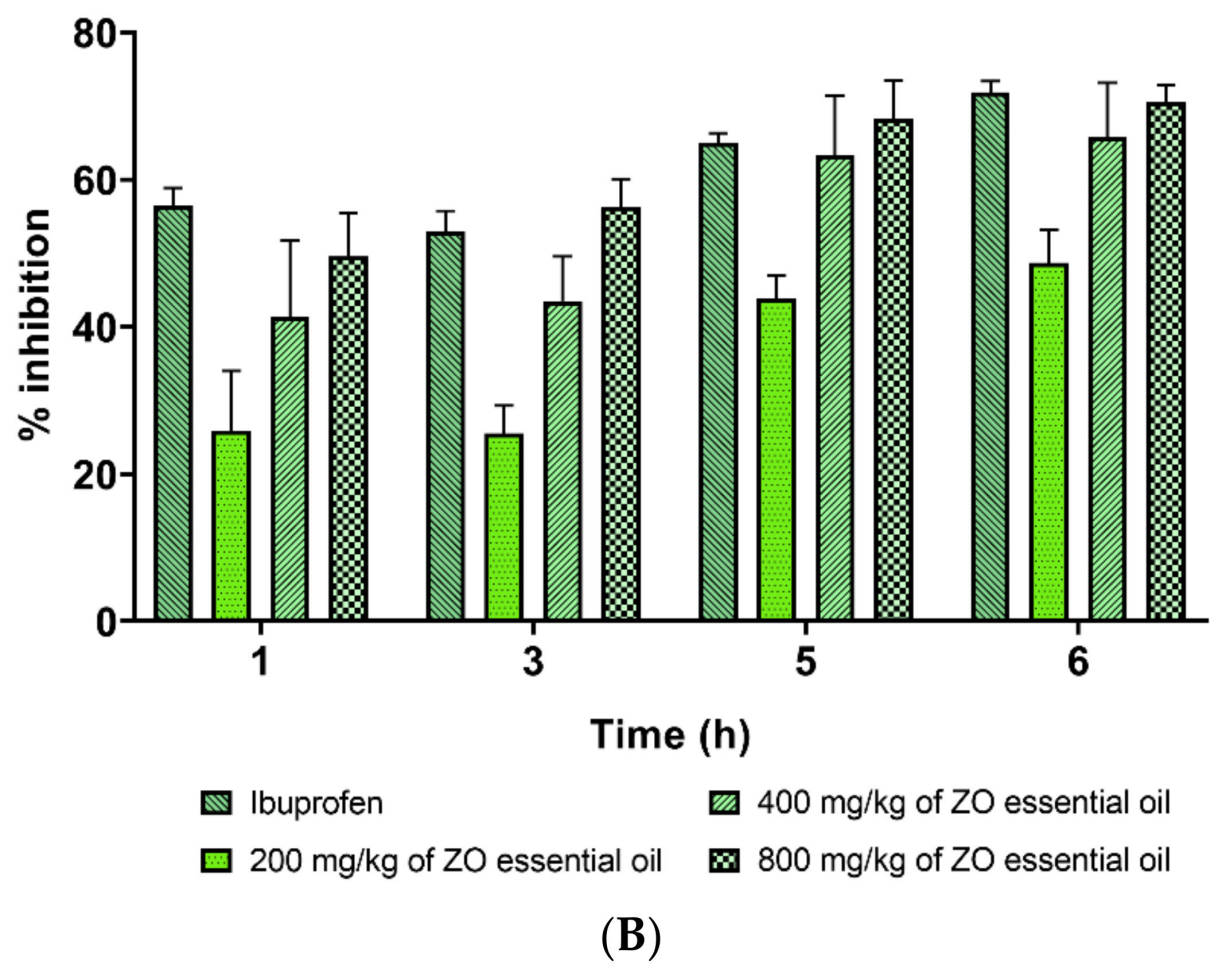
Effects of ZO essential oil and ibuprofen on carrageenan-induced hind paw edema in rats. (**A**) Mean changes in paw edema volume and (**B**) the percentages of paw edema inhibition after pretreating with ZO essential oil (200, 400, and 800 mg/kg) and ibuprofen (100 mg/kg) at different time points. Data represent the mean ± SEM (*n* = 6). * Denotes significant differences (*p* < 0.05) compared to the control group.

The effect of ZO essential oil on inflammation induced by carrageenan in the rat paw was revealed (Figure 2 and Figure 3). The gross pathological changes of paws of rats pretreated with ibuprofen (100 mg/kg) and ZO essential oil (200, 400, and 800 mg/kg), at 6 h after carrageenan injection, showed a reduction of paw edema when compared with the control group (Figure 2). In addition, both ZO essential oil (400 and 800 mg/kg) and ibuprofen significantly reduced hind paw edema when compared with the control group at 1, 3, 5, and 6 h after carrageenan injection. ZO essential oil at 200 mg/kg could also insignificantly reduce hind paw edema at 1 h after carrageenan injection (Figure 3A). The percentages of inhibition at 1, 3, 5, and 6 h after carrageenan injection of various doses of ZO essential oil and ibuprofen groups are shown in Figure 3B.

### 2.3. Effect of ZO Essential Oil on Carrageenan-Induced Hind Paw Edema in Rats: Histopathological Changes

**Figure 4:**
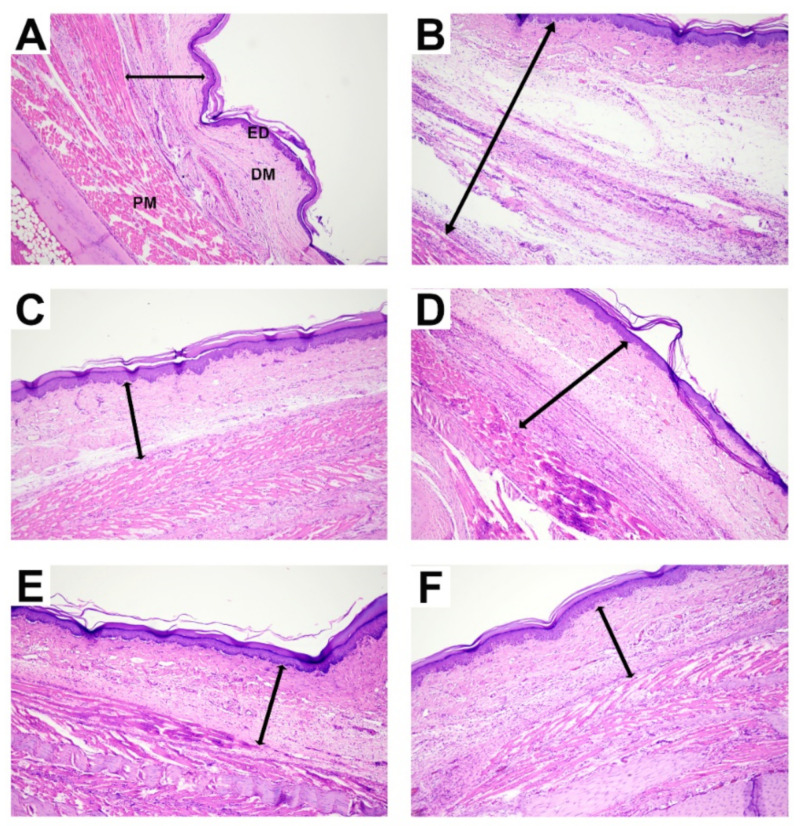
Histopathological examination of paw tissue of rats, pretreated with vehicle (0.9% saline), ibuprofen (100 mg/kg), or ZO essential oil at different doses, at 6 h after carrageenan injection. The tissue section from the paw tissue of each rat was stained with hematoxylin and eosin (100×). (**A**) Normal control group (no carrageenan injection); (**B**) control group; (**C**) ibuprofen group; (**D**–**F**) 200, 400, and 800 mg/kg of ZO essential oil groups. The arrow indicates the total thickness. Abbreviations: ED, epidermis; DM, dermis; PM; paw muscle.

The effect of ZO essential oil on inflammation induced by carrageenan in the rat paw, in terms of histopathological changes, is shown in Figure 4. The normal control group (no carrageenan injection) showed no significant edema formation and inflammatory cell infiltration in the paw tissue. Meanwhile, the control group showed moderate–severe inflammation, in terms of edema formation, loosening of connective tissues around the inflammatory sites, and the presence of inflammatory cell infiltration. On the other hand, the ibuprofen and ZO essential oil at 400 and 800 mg/kg groups showed only mild–moderate severity of inflammation, while ZO essential oil at 200 mg/kg showed moderate severity of inflammation.

### 2.4. Effect of ZO Essential Oil on Carrageenan-Induced Hind Paw Edema in Rats: Immunofluorescence Detection of COX-2 Expression

**Figure 5:**
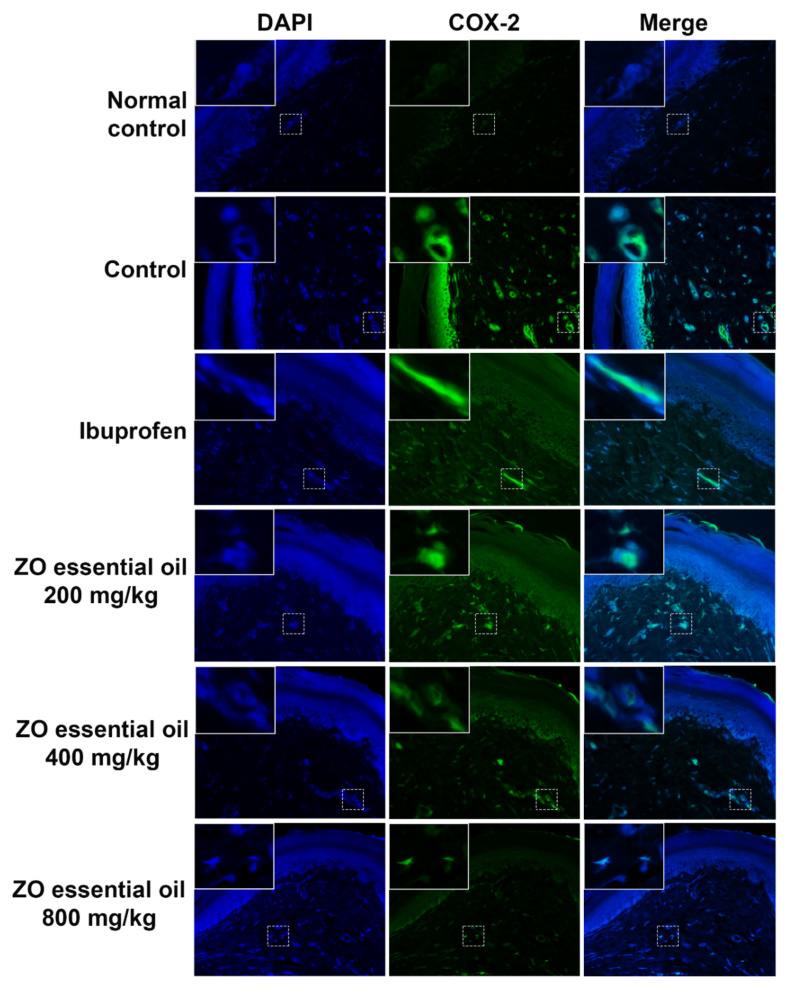
Immunofluorescence study for COX-2 expression in paw tissue of rats, pretreated with vehicle (0.9% saline), ibuprofen (100 mg/kg), or ZO essential oil at different doses, at 6 h after carrageenan injection. Micrographs were photographed by a fluorescence microscope (400× magnification). Abbreviations: DAPI, 4′,6-diamidino-2-phenylindole; COX-2, cyclooxygenase-2.

Determination of COX-2 expression in rat paw edema after inflammatory induction by carrageenan was investigated by immunofluorescence staining. The results showed no COX-2 expression in the normal control group (no carrageenan injection) (Figure 5). While COX-2 expression was found in the control and ZO essential oil (200 mg/kg) groups, the expression was reduced in the ibuprofen and other ZO essential oil (400 and 800 mg/kg) groups.

### 2.5. Effect of ZO Essential Oil on Carrageenan-Induced Hind Paw Edema in Rats: The PGE_2_ Level in Rat Serum

**Figure 6:**
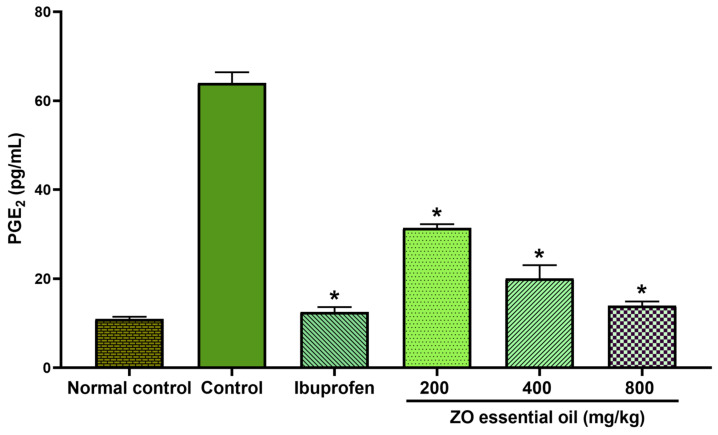
Measurement of PGE_2_ concentration in the serum of rats, pretreated with vehicle (0.9% saline), ibuprofen (100 mg/kg), or ZO essential oil at different doses, at 6 h after carrageenan. Data represent the mean ± SEM (*n* = 6). * Denotes significant differences (*p* < 0.05) compared to the control group.

Carrageenan injection into rat paw caused an increased PGE_2_ concentration in the serum. Serum PGE_2_ concentrations of the carrageenan-injected rats were significantly higher than those of the normal control group (no carrageenan injection) (Figure 6). The considerably lower PGE_2_ concentrations than the control rats were observed in the rats pretreated with ibuprofen and ZO essential oil. The alteration in serum PGE_2_ level appeared to occur in a dose-dependent manner. As the dose of ZO essential oil increased, the level of serum PGE_2_ became lower.

### 2.6. Effect of ZO Essential Oil on Carrageenan-Induced Hind Paw Edema in Rats: Immunofluorescence Detection of TNF-α Expression

**Figure 7:**
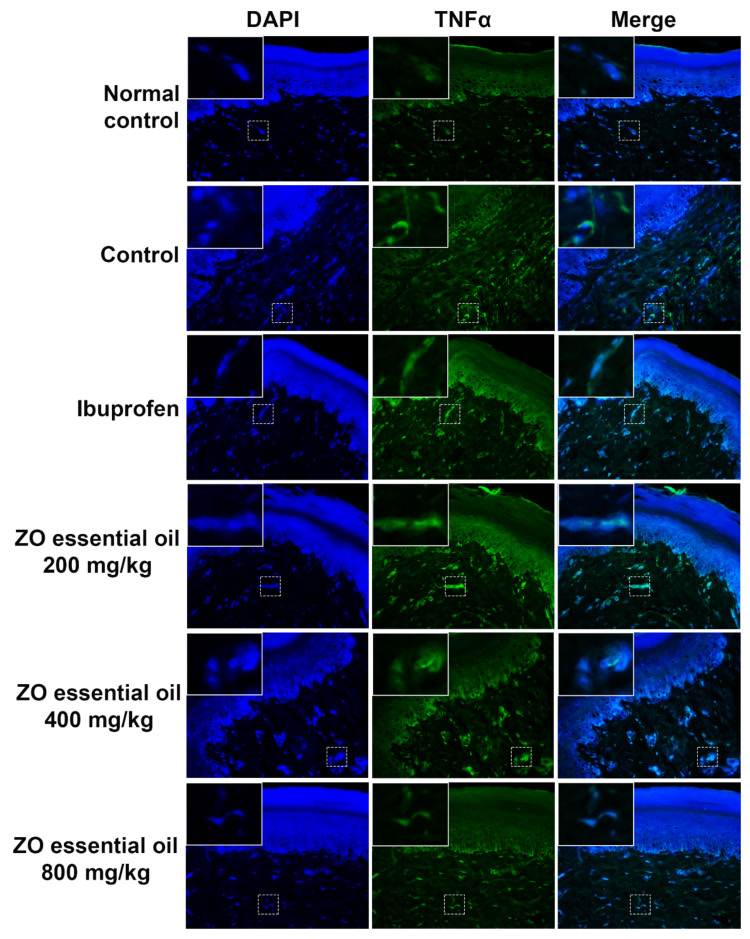
Immunofluorescence study for TNF-α expression in paw tissue of rats, pretreated with vehicle (0.9% saline), ibuprofen (100 mg/kg), or ZO essential oil at different doses, at 6 h after carrageenan injection. Micrographs were photographed by a fluorescence microscope (400× magnification). Abbreviations: DAPI, 4′,6-diamidino-2-phenylindole; COX-2, cyclooxygenase-2.

Determination of TNF-α expression in rat paw after inflammatory induction by carrageenan was investigated by immunofluorescence staining. The results showed no TNF-α expression in the normal control group (no carrageenan injection) (Figure 7). While TNF-α expression was found in the control and ZO essential oil (200 mg/kg) groups, the expression was reduced in the ibuprofen and other ZO essential oil (400 and 800 mg/kg) groups.

## 3. Discussion

Inflammation is part of the immunological host defense process against any agents, injuries, and stimuli that can lead to the production of various pro-inflammatory mediators associated with the development of many diseases if left unresolved within a short time [21]. Acute inflammation occurs quickly with noticeable inflammatory symptoms of swelling, heat, redness, pain, and infiltration of leukocytes into inflamed tissue [22]. Meanwhile, many pro-inflammatory mediators are released, such as IL-1β, IL-6, and TNF-α, as well as the expression of COX-2 and PGE_2_ [21,23]. The reduction of pro-inflammatory cytokines and mediators is very important to regulate the inflammatory responses. This present study determined the anti-inflammatory activity of ZO essential oil in rats by using two acute inflammatory models: EPP-induced ear edema and carrageenan-induced hind paw edema. EPP-induced ear edema is a common model used for screening the anti-inflammatory activity of any herbal extract and providing possible mechanisms of action [24]. EPP is an irritant agent that can induce redness and edema in rat ears by increasing vascular permeability, vasodilatation, and releasing pro-inflammatory mediators such as histamine, serotonin, kinins, and PGs [24]. ZO essential oil and ibuprofen significantly reduced rat ear edema compared to the vehicle control group. This result implies that ZO essential oil has an anti-inflammatory activity with a possible mechanism of action via inhibiting the release of the mentioned pro-inflammatory mediators.

Carrageenan is a chemical agent that can induce acute inflammation in rat paw, leading to edema, redness, and pain. The inflammatory processes involve the releasing of several mediators, depending on the phases of inflammation [5,25]. The first phase (a few hours after carrageenan injection) of the biphasic phase is mainly influenced by histamine, serotonin, and bradykinin from mast cells in the inflamed area and damaged tissue. The second phase (3–6 h after carrageenan injection) is mediated by arachidonic acid metabolites, mainly PGs, and various cytokines such as IL-1β, IL-6, TNF-α, and IL-10 [25,26]. Carrageenan-induced rat hind paw edema is another widely used animal model for evaluating the anti-inflammatory activity of plant extracts [25].

In this study, the oral administration of ZO essential oil (at all doses) and ibuprofen caused reduction of paw edema as shown in the gross pathological examination (Figure 2). The significant effect of ZO essential oil and ibuprofen in reducing paw edema was confirmed when considering the mean changes in paw edema volume and the percentages of paw edema inhibition (Figure 3). In addition, histopathology of paw tissue in the ZO essential oil and ibuprofen groups showed evidence of only mild–moderate severity of inflammation. The pronounced effect of ZO essential oil and ibuprofen was found during the second phase of inflammation (3–6 h after carrageenan injection), which is correlated mainly with the elevation of inducible COX-2 activity and the synthesis of PGE_2_ leading to vasodilation and increased vascular permeability in the rat paw [25,27]. Pre-treatment with ZO essential oil at 400 and 800 mg/kg and ibuprofen exhibited decreased COX-2 expression in paw tissue compared to the control group (Figure 5). Meanwhile, PGE_2_ levels in serum were significantly reduced in rats pre-treated with ZO essential oil at 200, 400, and 800 mg/kg and ibuprofen. As expected, ibuprofen (reference drug) is a medication in NSAIDs class targeting COXs inhibition [5].

Additionally, the second phase of carrageenan-induced inflammation in rat paw is also involved with the cytokine network products including TNF-α, a crucial mediator released from mononuclear phagocytes that causes edema formation, neutrophil migration, allodynia, immune response, and helps to increase the extension of a local or systematic inflammatory process [25,28,29]. Pre-treatment with ZO essential oil at 400 and 800 mg/kg and ibuprofen could reduce TNF-α expression in rat paws. Furthermore, it was found that protein from rhizomes of ZO could inhibit pro-inflammatory cytokines, including IL-6 and TNF-α, in the macrophage cell line [20]. In addition, ZO essential oil was reported to decrease IL-6 levels in Hela cells [19]. IL-6 significantly affects the production of TNF-α (positive feedback), and IL-6 amplifies the expression of COX-2 protein and activity that subsequently enhances the formation of PGE_2_ [30]. From our previous study about the phytochemical profile of ZO essential oil, results revealed that ZO essential oil had been found to contain sesquiterpene and monoterpene compounds including zerumbone (24.73%), terpinene-4-ol (18.75%), and sabinene (15.19%) [15]. Notably, zerumbone and terpinene-4-ol are fascinating natural compounds reported to manifest a broad spectrum of pharmacological activities, e.g., anti-inflammation, antioxidant, and anticancer, in many studies [19,31,32]. Furthermore, sabinene also exhibited anti-inflammatory activity [33]. According to all of this previous information, together with the results from the carrageenan-induced inflammatory model, it is reasonable that ZO essential oil possessed anti-inflammatory activity. Its possible mechanisms of action involved the inhibition of pro-inflammatory cytokine TNF-α expression as well as the inhibition of COX-2 and PGE_2_ productions.

## 4. Materials and Methods

### 4.1. Plant Materials and Extraction of Zingiber ottensii (ZO) Valeton Essential Oil

*ZO* was planted in Mae Rim District, Chiang Mai, Thailand. Its rhizomes were harvested in March 2020. Plant identification was performed at the Faculty of Pharmacy, Chiang Mai University (voucher specimen 000109). The ZO essential oil was further extracted from fresh rhizomes using the same method as described in a previous study [15].

### 4.2. Laboratory Animal Care and Maintenance

Male Wistar rats (*Rattus norvegicus*) (40–60 g and 100–120 g) were purchased from Nomura Siam International Co. Ltd., Bangkok, Thailand. All animals were acclimatized for a week before experiments in an animal room maintained under standard conditions (temperature at 24 ± 1 °C, 50 ± 10% relative humidity, and a 12:12 h light–dark cycle. They had free access to a standard pelleted diet and drinking water. The Animal Ethics Committee of the Faculty of Medicine, Chiang Mai University, Thailand, approved all experiments (permit no. 22/2563, 9 July 2020).

### 4.3. Ethyl Phenylpropiolate-Induced Rat Ear Edema

Anti-inflammatory activity of ZO essential oil was screened according to the method described in the previous study, with slight modifications [24]. Male Wistar rats (40–60 g) were randomly divided into three equal groups, three rats (six ears)/group. The rat ear edema was induced by topical application of EPP 50 mg in 1 mL of acetone (1 mg/20 µL/ear) on both ears’ inner and outer surfaces. ZO essential oil (3 mg/ear) and ibuprofen (1 mg/ear) (both diluted in acetone) and acetone (vehicle) were applied in the same manner at a volume of 20 µL/ear before the application of EPP.

The ear thickness was measured with a digital Vernier caliper at 0, 15, 30, 60, and 120 min after EPP application. The increase in ear thickness was compared with that of the vehicle-treated group, and the percent inhibition was calculated.

### 4.4. Carrageenan-Induced Rat Hind Paw Edema

Anti-inflammatory activity was performed according to the method described in the previous study, with slight modifications [24]. Male Wistar rats (100–120 g) were randomly divided into six equal groups (six rats for each group). The rats were pre-treated orally with 0.9% saline (vehicle), ibuprofen (100 mg/kg), or ZO essential oil (200, 400, or 800 mg/kg) diluted in 0.9% saline. After 1 h, acute inflammation was produced by subplantar injection of carrageenan (1% *w*/*v* in sterile normal saline, 0.05 mL/paw) into the right hind paw of rats, except in the normal control group. The paw edema was measured using a volume displacement technique with a plethysmometer (model 7140, Ugo Basile, Italy) at 0, 1, 3, 5, and 6 h after carrageenan injection. The edema volume of the paw and the percent edema inhibition of each test compound were calculated. Six hours after carrageenan injection, the rats were sacrificed. The blood samples were collected from rats and kept in a clot blood tube overnight at 4 °C before centrifugation for 15 min at 1000 rpm. Serum was isolated and stored at −20 °C of PGE_2_ assay. The right hind paw was dissected and fixed in 10% neutral buffered formalin and then placed in the decalcifying agent (10% formic acid; Sigma-Aldrich, Saint Louis, MO, USA).

### 4.5. Determination of PGE_2_ Level in Rat Serum

According to the manufacturer’s instruction, the concentration of PGE_2_ in serum was measured using Rat PGE_2_ ELISA Kit (CSB-E07967r; Cusabio Biotechnology, Wuhan, China). Absorbance was measured immediately at 450 nm using a microplate reader (BioTek Instruments, Winooski, VT, USA). The level of PGE_2_ was expressed as picograms per milliliter of serum.

### 4.6. Histopathological Examination of Rat Paw Tissue

The right hind paw of rat was removed from the decalcifying agent, rinsed, dehydrated in a graded series of ethanol, and then embedded in paraffin wax. After that, tissues were cut into a five-micrometer section, deparaffinized, and then stained with hematoxylin and eosin (H&E; Sigma-Aldrich, Saint Louis, MO, USA). The histopathological changes and inflammatory response in paw tissue were examined under a microscope. According to the previous study, the degree of inflammation was evaluated and blindly described by two veterinary pathologists at Veterinary Diagnostic Center, Chiang Mai University [5,34,35].

### 4.7. Immunofluorescence Study for COX-2 and Pro-Inflammatory Cytokine TNF-α Expression in Rat Paw Tissue

Immunofluorescence staining was performed using a previously described method, with slight modifications [36]. Briefly, the paraffin boxes were cut into a four-micrometer section, deparaffinized, and dehydrated with xylene (Labscan Asia, BKK, Thailand) and ethanol (VMR international, Fontenay-sous-Bois, France). Then, antigens were unmasked using a citrate-based antigen retrieval solution (Vector Laboratories, Burlingame, CA, USA) and heated to boiling. Then, the slides were washed twice for 2 min with phosphate buffered saline (PBS) and once for 10 min with PBS and subjected to permeabilization by using a permeabilization solution (0.2% gelatin, 0.25% triton-x in PBS) twice for 10 min. The slides were then blocked with 5% bovine serum albumin (BSA) in permeabilization solution for 1 h before being incubated with specific antibodies. The primary antibodies (rabbit anti-COX-2 antibody, Cell Signaling Technology, Danvers, MA, USA; or rabbit anti-TNF-α antibody, Biotium, Fremont, CA, USA) were diluted in 1% BSA in permeabilization solution and then added to each slide and incubated overnight. The slides were washed twice with PBS for 10 min, once for 10 min with permeabilization solution, and incubated for 2 h at room temperature in 1% BSA with secondary antibodies Cy3- or FITC-conjugated goat anti-rabbit IgG (ABclonal, Woburn, MA, USA). Furthermore, the nuclei were stained with 1 μg/mL of 4′,6-diamidino-2-phenylindole (DAPI) (Sigma-Aldrich, Saint Louis, MO, USA) for 2 h in the dark at room temperature. After final washing with PBS 4 times for 10 min and once with distilled water, the slide sample was mounted with Vectashield (Vector Laboratories, Burlingame, CA, USA) and coverslip. The observations were performed on a fluorescence microscope, Axio Vert. A1 (Carl Zeiss, Oberkochen, Germany), with 400× magnification. Micrographs were captured with the Zen2.6 (blue edition) Software for the Zeiss Axiocam 506 color microscope camera.

### 4.8. Statistical Analysis

The results were compared by means of one-way analysis of variance (ANOVA) and Tukey’s multiple comparison test using SPSS Statistical Package version 22 (IBM, Armonk, NY, USA), and the figures were generated using GraphPad Prism8 software (San Diego, CA, USA). Data were expressed as mean ± SEM. Statistical significance of differences was set at *p* < 0.05.

## 5. Conclusions

The essential oil of *Zingiber ottensii* Valeton possessed anti-inflammatory activity in both animal models, the EPP-induced ear edema and carrageenan-induced paw edema in rats. Its possible mechanisms of action involved the inhibition of pro-inflammatory cytokine TNF-α expression as well as the inhibition of COX-2 and PGE_2_ production. These preclinical findings provide more crucial data about the anti-inflammatory activity of ZO essential oil that strengthen its possibility to be developed into new herbal health products/medicines as alternative ways for anyone who denies using modern medicines in the future. Further investigation of its precise mechanism of action and chronic toxicity study is warranted before clinical application.

## Data Availability

Not applicable.
